# Natural Compounds Targeting *SIRT1* and Beyond: Promising Nutraceutical Strategies Against Atherosclerosis

**DOI:** 10.3390/nu17213316

**Published:** 2025-10-22

**Authors:** Elisa Domi, Malvina Hoxha

**Affiliations:** Department for Chemical-Toxicological and Pharmacological Evaluation of Drugs, Faculty of Pharmacy, Catholic University Our Lady of Good Counsel, Rruga Dritan Hoxha, 1000 Tirana, Albania; m.hoxha@unizkm.al

**Keywords:** *SIRT1*, atherosclerosis, natural compounds, inflammation, oxidative stress, vascular protection

## Abstract

**Background/Objectives**: Atherosclerosis remains a leading cause of morbidity and mortality worldwide, with an urgent need for novel preventive and therapeutic strategies. Sirtuin 1 (*SIRT1*), an NAD^+^-dependent deacetylase, has emerged as a central regulator of vascular homeostasis, modulating oxidative stress, inflammation, lipid metabolism, and endothelial function. Increasing evidence highlights that some natural activators of *SIRT1* may be interesting in mitigating the development of cardiovascular diseases. **Methods**: Searching in the main databases PubMed and Scopus, we made a literature revision, including studies from January 2000 to June 2025, of the major natural *SIRT1* activators involved in vascular impairment in order to investigate their potential therapeutic use in atherosclerosis. **Results**: Among them, resveratrol, quercetin, naringenin, and hydroxytyrosol show the strongest evidence in activating *SIRT1* and modulating the essential molecular pathways involved in atherosclerotic disease. These findings span from preclinical to clinical studies, with limited randomized clinical trial data for hard cardiovascular outcomes. **Conclusions**: This review synthesizes current knowledge on natural *SIRT1* activators in the context of atherosclerosis, emphasizing their molecular mechanisms and clinical perspectives. The concept of using nutraceuticals-based interventions targeting *SIRT1* may pave the way for innovative strategies in cardiovascular diseases.

## 1. Introduction

Aging is a natural process of physiological decline that contributes to the pathogenesis of cardiovascular diseases (CVDs), including atherosclerosis. Atherosclerosis represents a leading cause of morbidity and mortality worldwide, with an urgent need for novel preventive and therapeutic strategies.

The early stage of this pathology is characterized by endothelial damage, where an altered endothelium-dependent vasorelaxation is observed. This step is generally a result of unbalanced nitric oxide (NO) production by endothelial NO synthase (*eNOS*) and a defective signaling pathway, leading to increased oxidative stress, inflammation, and impaired autophagy [1]. The progression of the disease is followed by the mobilization and the permeation of immune cells like monocytes/macrophages, foam cells, lipoproteins, inflammatory factors, necrotic cell debris, and fibrous components [1]. All these events, in addition to contributing to inflammatory and oxidative processes, promote the development of atherosclerotic lesions in the arterial walls, which are the main characteristic of atherosclerosis [1,2]. Vascular aging does not only include endothelial cells but also vascular smooth muscle cells (VSMCs). Indeed, damaged structure and functionality of these cells may take part in the progression of this disease [1].

The onset of atherosclerosis is a complex and progressive process, mainly controlled by the vascular endothelium function and its ability to counteract different stress stimuli [3]. In turn, the vascular endothelium function is affected by several risk factors that are not yet fully understood; however, among the main known causes are genetic predisposition, diabetes mellitus, obesity, hypertension, and bad lifestyle habits like smoking, unbalanced diet, and sedentary lifestyle [4]. Also, an impaired lipid metabolism, characterized by increased levels of total cholesterol (TC) and low-density lipoprotein (LDL) and reduced levels of high-density lipoprotein (HDL), plays a crucial role in this disease [4]. The main consequences deriving from atherosclerosis are angina, due to reduced blood flow in the arterial lumen; myocardial infarction, which may occur as a result of lesion rupture and blood flow obstruction; and stroke due to obstruction of cerebral arteries [2].

Given the danger and the high incidence of the disease, there is a great interest in finding different approaches for preventing and eventually treating atherosclerosis. One of the most targeted mechanisms is associated with endothelial senescence.

In this regard, sirtuin1 (*SIRT1*) plays a pivotal role as a longevity and cell survival regulator. It is involved in several functions, including senescence, inflammatory processes, and oxidative stress. *SIRT1* belongs to a class of nicotinamide adenine dinucleotide (NAD^+^)-dependent histone deacetylases and under stressful stimuli, when NAD^+^ levels are increased, its transcription is activated [5]. Once *SIRT1* is directly or indirectly activated, it deacetylates its target proteins, found in both the nucleus and cytoplasm. The deacetylation process leads to a stimulation of the nuclear factor erythroid 2-related factor 2/antioxidant response element (*Nrf2*/ARE) pathway, which in turn promotes the expression of some antioxidant enzymes like superoxide dismutase (*SOD*), catalase (*CAT)*, and glutathione peroxidase (*GPx*). In addition, *SIRT1* suppresses the transcription of pro-inflammatory genes by modulating the nuclear factor kappa B (NF-κB) pathway, which contributes to the progression of inflammatory diseases, including atherosclerosis [6,7]. Moreover, *SIRT1* activation promotes the modulation of some key proteins like tumor suppressor p53, hypoxia-inducible factor 1-alpha (*HIF-1α*) and forkhead box O (FOXO) family of transcription factors, peroxisome proliferator-activated receptor gamma (*PPARγ*), the cofactor of *PPARγ* (*PGC-1α*), and Ku70 [6]. Regarding *SIRT1* activation, we distinguish between direct activation, which is promoted by compounds that physically interact with *SIRT1* protein, either by binding to its allosteric site or catalytic domain, thereby enhancing its deacetylase activity, and indirect activation, resulting from compounds that modulate upstream pathways influencing *SIRT1* activity, expression, or cofactor availability. These mechanisms include, for example, the increase in the cellular NAD^+^/NADH ratio, the upregulation of *SIRT1* transcription, and the decrease in oxidative and inflammatory stress, thereby preventing post-translational inhibition of *SIRT1* [5].

All these mechanisms suggest an interesting role of *SIRT1* activation in the improvement and progression of endothelial alteration, vascular aging, and age-related CVDs.

Therefore, relying on the different pathways mentioned above, the use of *SIRT1* activators has aroused interest as potential therapeutic agents that can be used in the therapy and especially in the prevention of atherosclerosis.

In our study we wanted to focus on the implementation in our diet of some natural activators, found in different fruits, vegetables, and plants, targeting the importance of a nutraceutical approach as a new therapeutic strategy to improve the disease.

## 2. Materials and Methods

This review was performed to summarize and discuss the current state of knowledge of natural compounds with relevance to atherosclerosis and potential modulation of *SIRT1*.

### 2.1. Literature Search Strategy

The literature survey was performed using PubMed and Scopus as main databases, and the main terms searched were as follows: “*SIRT1* and atherosclerosis”, “natural compounds and atherosclerosis”, “nutraceuticals and atherosclerosis”, “polyphenols and atherosclerosis”, “flavonoids and atherosclerosis”, “oxidative stress and atherosclerosis”, “inflammation and atherosclerosis”, “lipid metabolism and atherosclerosis”, “vascular protection and atherosclerosis”, “natural compounds and *SIRT1*”, “nutraceuticals and *SIRT1*”, “polyphenols and *SIRT1*”, “flavonoids and *SIRT1*”, “oxidative stress and *SIRT1*”, “inflammation and *SIRT1*”, “lipid metabolism and *SIRT1*”, and “vascular protection and *SIRT1*”.

### 2.2. Elegibility Criteria

In our review we included original experimental studies and review articles addressing the role of some natural compounds, which potentially modulate *SIRT1* activity and prevent or slow atherosclerosis through different mechanisms. We excluded studies not related to cardiovascular outcomes, articles lacking molecular or mechanistic data, and articles including bioactive compounds that do not involve *SIRT1* modulation. So, to summarize, the included compounds have been chosen based on the following criteria: (I) availability of evidence in models of atherosclerosis; (II) involvement of *SIRT1* modulation, both directly and indirectly, and additional pathways relevant to endothelial function, oxidative stress, lipid metabolism, and inflammation. On this basis we selected resveratrol, quercetin, naringenin, curcumin, berberine, fisetin, piceatannol, honokiol, epigallocatechin-3-gallate, and hydroxytyrosol. The data were categorized based on the compound studied and its mechanisms of action. Only English language articles were considered.

### 2.3. Study Selection

**Figure 1:**
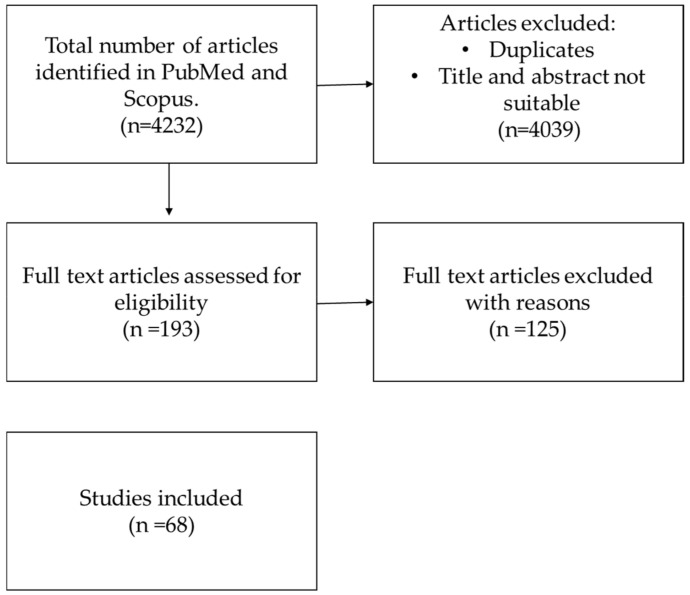
Prisma flow diagram represents the steps we followed during the literature search and article selection for this systematic review.

From PubMed and Scopus databases, a total of 4232 articles were initially retrieved. First of all, we excluded 4039 articles that were duplicates and studies whose titles and abstracts did not meet the chosen criteria. As a result, 193 articles were assessed for a full-text analysis, and only 68 papers were included in this review, as they met the criteria. The PRISMA diagram below shows the steps followed during our literature search. (Figure 1).

### 2.4. Data Extraction

We identified four thousand two hundred thirty-two papers, of which sixty-eight were finally included in this systematic review.

## 3. Results

### 3.1. SIRT1 and Atherosclerosis

#### 3.1.1. *SIRT1* and Aging

*SIRT1* is highly expressed in the vascular tissue, where it regulates its functions during vascular growth and angiogenesis [8]. Moreover, it is observed that a low *SIRT1* expression is associated with a more rapid process of senescence in endothelial cells [9]. The mechanism implicated in the preventive effect of *SIRT1* in endothelial aging is attributed to the deacetylation of the liver kinase B1 (*LKB1*), which, once it is deacetylated, undergoes degradation. Conversely, during aging, the loss of *SIRT1* activity promotes the accumulation of the acetylated form of *LKB1* in the nucleus of endothelial cells, leading to a structural and functional remodeling of the arterial walls [9].

During aging, the expression level of *SIRT1* in VSMCs is progressively reduced; in addition, its downregulation, promoted by vascular miR-34a (a micro-RNA involved in post-transcriptional regulation of gene expression), promotes inflammation and vascular aging. Overall, these mechanisms confirm the key role of *SIRT1* in vascular senescence [10,11].

#### 3.1.2. *SIRT1* and *eNOS*/NO

Endothelial *SIRT1* seems to regulate the production of NO, an endogenous gasotransmitter synthesized by *eNOS*, which promotes vascular homeostasis and vascular tone, showing anti-atherosclerotic and anti-aging properties [12]. In fact, it is observed that in the endothelium of aged mice there are reduced levels of *SIRT1* expression in relation to younger mice; moreover, in aged mice in relation to the younger ones, there is a prevalence of the inactive acetylated form of *eNOS* [13].

To confirm the beneficial effect of *SIRT1* in vasodilation, it has been observed that a depletion of *SIRT1* in the endothelium leads to a reduced production of endogenous NO and a lack of endothelium-dependent vasodilation [14]. The regulation of *eNOS* by *SIRT1* activation occurs at both transcriptional and post-transcriptional stages, promoting the production of NO. Furthermore, the generated NO also enhances the expression of *SIRT1* in endothelial cells, although this mechanism is not yet fully understood [12].

In its favor, there is a study in which it was observed that the use of a phosphodiesterase 3 inhibitor (cilostazol) contained the metabolic stress triggered by an early aging of endothelial cells. In fact, using this inhibitor, the enzyme *eNOS* remains in its active and phosphorylated form by cyclic adenosine monophosphate (cAMP)/protein kinase A (*PKA*). The stimulation of *eNOS* therefore promotes the production of NO, which in turn favors the upregulation of *SIRT1*, thus suggesting the existence of the *eNOS*/NO/*SIRT1* axis involved in the protection of the endothelium and as a result of atherosclerosis [15].

#### 3.1.3. *SIRT1* and Inflammation

Inflammation is the key event that triggers the development of atherosclerosis and involves both endothelial and immune cells like monocytes and macrophages. At these levels, inflammation leads to chronic activation of NF-κB, consequently resulting in the upregulation of pro-inflammatory mediators like vascular adhesion molecule-1 (VCAM-1), monocyte chemoattractant protein-1 (MCP-1), inducible nitric oxide synthase (*iNOS*), cyclooxygenase -2 (*COX-2*), tumor necrosis factor α (*TNFα*), interleukin-6 (IL-6), and interleukin-1β (IL-1β) [16]. In conclusion, this inflammatory state contributes to endothelial dysfunction.

*SIRT1* exerts its protective role through the deacetylation of the *NF-κB* p65 subunit; the deacetylated factor is no longer able to promote the expression of pro-inflammatory signal molecules, resulting overall in a reduced inflammation state. On the contrary, it has been observed that the suppression of *SIRT1* leads to an increased rate of acetylated *NF-κB*, thus promoting vascular inflammation [17,18]. It has been demonstrated that patients with coronary artery disease show lower *SIRT1* mRNA expression and higher IL-6 expression in blood monocytes versus healthy controls [19].

Moreover, it has been observed that patients affected by both coronary artery disease and type 2 diabetes show an inverse correlation between the increase in inflammatory cytokines and *SIRT1* upregulation in peripheral blood mononuclear cells [20]. In fact, in patients with metabolic disorders, it has been observed that insulin resistance leads to reduced levels of *SIRT1* [21]. On the contrary, caloric restriction in obese patients leads to an upregulation of *SIRT1* with a consequent reduction in inflammation markers [22]. These mechanisms confirm the importance of *SIRT1* in modulating inflammation in atherosclerosis, but not only in conditions that favor its onset, such as diabetes and obesity.

#### 3.1.4. *SIRT1* and Oxidative Stress

Oxidative stress is generated when there is an imbalance between pro-oxidative enzymes, which trigger an overproduction of reactive oxygen species (ROS), and the stimulation of anti-oxidative molecules. This condition over time leads to endothelial impairment, resulting in the senescence of blood vessels, thus atherosclerosis [23].

*SIRT1* is involved in anti-oxidative processes through several mechanisms, including the repression of p66Shc protein transcription. This protein has oxidoreductase activity; in fact, it stimulates the production of ROS by the mitochondria. *SIRT1*, by inactivating p66Shc, plays a protective role against age-related endothelial dysfunction, and, consequently, atherosclerosis [24]. Another antioxidant mechanism proposed for *SIRT1* involves the deacetylation of some antioxidant enzymes in endothelial cells like manganese *SOD* (*Mn-SOD*), *CAT*, peroxiredoxins 3 and 5 (Prx3, Prx5), thioredoxin 2 (Trx2), and thioredoxin reductase 2 (*TR2*). In addition, *SIRT1* promotes FOXO3 transcription factor activation and *PGC-1α* stimulation in endothelial cells, resulting in antioxidant activity [25].

It is observed that in atherosclerosis there is a reduced expression of *SIRT1* in VSMCs, and this deficiency is associated with increased oxidative stress processes and oxidized low-density lipoprotein (oxLDL), which in turn leads to DNA damage in vascular tissues [26]. The progressive vascular damage leads to further ROS overproduction, which in turn activates the *NF-κB* factor, triggering chronic inflammation. As a result, there is a constant inflammatory and oxidative state where *SIRT1* may play a key role in breaking this vicious cycle.

#### 3.1.5. *SIRT1* and Autophagy

Autophagy is an essential mechanism needed for the preservation of vascular homeostasis, as it degrades damaged molecules that could alter vascular tissue. During cellular aging, this mechanism is impaired, triggering degradation processes that promote vascular injury then atherosclerosis [27]. *SIRT1* promotes autophagy in the nucleus and in the cytoplasm as well by being involved in the different stages of this process, including initiation, prolongation, maturation, fusion, and degeneration. In the nucleus, *SIRT1* promotes autophagy mostly through the deacetylation of FOXO1 and FOXO3 [28], while in the cytoplasm, through some autophagy factors, ATG5, ATG7, and ATG12, which are substrates of deacetylation [29].

In the initiation step, *SIRT1* regulates autophagy through the maintenance of Tuberous Sclerosis Complex 2 (TSC2) stability. Then TSC2 downregulates mammalian target of rapamycin complex 1 (mTORC1), finally promoting autophagy [30].

In case of excess nutrition, the autophagy process involves the axis AMP-activated kinase (*AMPK*)/*SIRT1*/mTORC1. In fact, overnutrition is followed by an increase in mTORC1 levels, which leads to decreased *SIRT1* and *AMPK* levels, resulting in autophagy inhibition. Conversely, when *AMPK* is activated, it promotes the production of NAD^+^, which successively activates *SIRT1*; on the other hand, *SIRT1* activates *AMPK* by the deacetylation and consequent activation of *LKB1* [31].

Regarding elongation and maturation steps, *SIRT1* regulates autophagy processes through different mediators. Under excess nutrition, the acetylation of ATG5, ATG7, and ATG12 by E1A binding protein p300 acetyltransferase (*EP300*) blocks the generation of the complex ATG16-ATG5-ATG12, which leads to the prevention of autophagosome elongation [31]. Meanwhile, in food deprivation conditions, *SIRT1* promotes the generation of the complex ATG16-ATG5-ATG12 through the deacetylation of ATG5, ATG7, and ATG12. As a result, this leads to the elongation of autophagic vesicles [32]. Moreover, the elongation of the autophagosome is controlled by *SIRT1* also by regulating the translocation of nuclear microtubule-associated protein 1 light chain 3 (MAP1LC3-I) to the cytoplasm, where it contributes to the formation of LC3-II [33].

Finally, *SIRT1* also exerts its pro-autophagic role by mediating the fusion and, consequently, the degradation process. Especially in starvation, *SIRT1* promotes FOXO1 deacetylation, which consequently results in the activation of the factor Rab7 that facilitates the fusion of the mature autophagosome to the lysosome [34].

**Figure 2:**
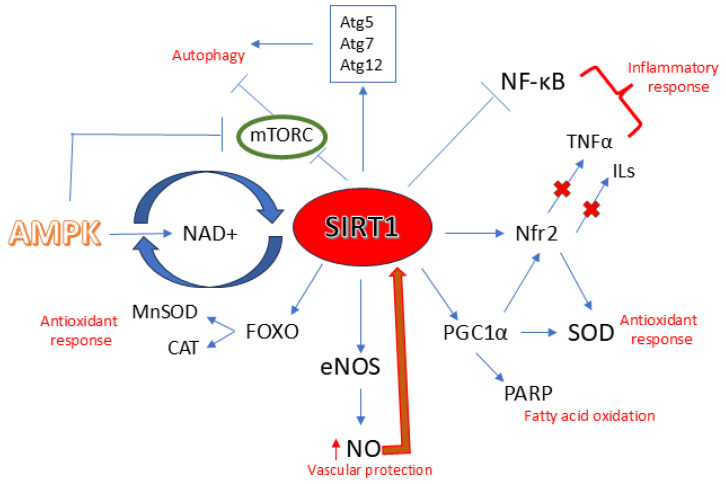
Schematic representation of the mechanisms promoted by *SIRT1* involved in atherosclerosis. As observed in the figure, the enzyme acts on multiple pathways, making it an interesting target.

Therefore, relying on the different pathways mentioned above, *SIRT1* seems to be an interesting target for atherosclerosis (Figure 2).

### 3.2. Natural Activators and Atherosclerosis

Given the above-mentioned mechanisms of antiatherogenic activity of *SIRT1*, it is interesting to evaluate the importance of its activators as a potential therapeutic approach. In particular, in recent years, the emphasis on a healthy and balanced diet has increased as a good lifestyle habit for the prophylaxis of various diseases. In this regard, the long-term use of some bioactive compounds with antioxidant and anti-inflammatory properties, seems to avoid or decelerate the course of cardiovascular diseases [35]. Natural activators of *SIRT1*, found mainly in plant foods, fruits, and vegetables, are in fact the molecular target that we have chosen to summarize in this review as potential beneficial agents in atherosclerosis [36]. In Table 1, we list the natural activators of *SIRT1* along with a summary of their main mechanisms of action against atherosclerosis.

#### 3.2.1. Resveratrol

Resveratrol (RSV) (3,5,4′-trihydroxystilbene) is a natural polyphenol identified in 72 species of plants, especially grapes, peanuts, and berries, and it is also present in red wine [37]. It is found in two isomeric forms, *cis* and *trans*, where the *trans* one can be transformed to *cis* under heated conditions [38]. RSV supplementation in humans typically ranges from 150 to 500 mg/day in clinical trials, while dietary sources and wine provide much lower amounts (5–10 mg/day). After oral intake, plasma concentrations of free RSV are usually in the range of 0.1–0.5 μM, with most circulating as glucuronide and sulfate conjugates, which are the main metabolites. In contrast, many in vitro studies demonstrate that *SIRT1* activation uses concentrations between 5 and 50 μM, which exceed those achieved in vivo. Nevertheless, RSV metabolites may exert biological activity, and also to increase RSV bioavailability, it may be suggested to adopt some strategies that include the following: coadministration with piperine or quercetin (QRC); acetylation of the hydrophilic hydroxyl groups, which represent the target site of sulfation and glucuronidation; and nanoformulations [38].

RSV is noted for its anti-aging, antioxidant, and anti-inflammatory properties; also, it regulates lipid metabolism, blood pressure, cell adhesion, and endothelial function. For these reasons it plays an interesting role in cardiovascular diseases, including atherosclerosis [39].

It is demonstrated that oral consumption of high doses of RSV seems to be safe; in fact, when 5 g of RSV was ingested daily by humans for 29 consecutive days, peak plasma concentrations of *trans*-resveratrol reached up to 4.2 μM, with no associated risks or side effects; thus, its use may be interesting [40]. However, several clinical studies reported that RSV is characterized by low bioavailability due to the rapid metabolism of the isomer *trans*-RSV, which, even if it has a superior bioactivity, is rapidly metabolized into glucuronide and sulfate conjugates [40].

RSV enhances *SIRT1* activity both directly (via allosteric modulation) and indirectly by increasing NAD^+^ levels and activating *AMPK*. Also, by promoting *SIRT1* activity, RSV suppresses inflammation and oxidative stress through the deacetylation of p53, *NF-κB*, and FOXO transcription factors [41].

The anti-atherosclerotic effect derived from RSV supplementation is demonstrated in different animal models and vascular cells and exerts its properties at different levels through different mechanisms.

First of all, it is observed that it can reduce the size of atherosclerotic lesions, promoting the regulation of vascular structure. In atherosclerosis, VSMCs contribute to plaque formation by migrating to the endothelium, where they multiply and produce an extracellular matrix, also promoting platelet coagulation. RSV was shown to mitigate platelet coagulation through the following different mechanisms: (a) inhibition of platelet adhesion to the collagen (10–1000 μM of RSV in vitro using blood from healthy humans; 4 mg/kg daily in vivo in rabbits); (b) inhibition of the influx of Ca^2+^ into thrombin-stimulated platelets (0.1, 1.0, and 10.0 μM of *trans*-resveratrol in human platelets); (c) reduction in tissue factor (*TF*) activity, which contributes to blood coagulation (10 to 100 μM of RSV in human peripheral blood monocytes and monocytic cell line, THP-1) [42,43,44]. All these effects are related to the inactivation of the phosphoinositide 3-kinase (*PI3K*)/protein kinase B (*Akt*)/mammalian target of rapamycin (mTOR) pathway. Moreover, RSV downregulates the expression of the matrix metalloproteinase-9 (*MMP-9*), resulting in reduced vascular remodeling [45].

Concerning lipid metabolism, it has been observed that the use of 50 mg/kg/day of RSV in apolipoprotein E-deficient (ApoE^−/−^) mice and umbilical vein endothelial cells (UVECs) isolated from these mice helped in the reduction in TC, triglycerides (TG), and low-density lipoprotein cholesterol (LDL-C) levels and led to a mild increase in high-density lipoprotein cholesterol (HDL-C) levels. Also, its use is associated with a decrease in TNF-α, C-reactive protein (CRP), and CD40L expression levels in arterial lesion tissue. The mechanism underlying these effects seems to be due once again to the modulation of the axis *PI3K*/*Akt*/mTOR [46].

The deposition of LDL-C in vascular walls promotes the formation of atherosclerotic plaques, suggesting this molecule as a key target to prevent the disease. Low-density lipoprotein receptor (LDLR) degrades around 75% of plasma LDL-C, promoting the clearance of endogenous cholesterol, and RSV exerts its beneficial effect by upregulating LDLR expression [47].

Also, RSV seems to protect the peroxidative degradation of lipids in the vascular wall. The mechanism behind this effect is due to the protection of LDLs against ferromyoglobin and peroxynitrite, two potent oxidative molecules (2, 4, or 6 μM of RSV in human LDL particles) [48].

It has been demonstrated that RSV may improve atherosclerosis, also influencing the recruitment and the adherence of circulating blood monocytes. In fact, it has been observed that the use of 20 mg/kg of RSV reduces the expression and the levels of several chemokines and adhesion molecules, like chemokine (C-C motif) ligand 2 (CCL2), chemokine (C-X-C motif) ligand 1 (CXCL1), intercellular adhesion molecule-1 (ICAM-1), and VCAM-1, both in human endothelial cells and in THP-1 human monocytes [49].

As mentioned before, the crucial role of RSV in atherosclerosis is also due to its anti-inflammatory properties; in fact, it acts by preventing the damage to elastin fibers induced by TNF-α—induced in aortic tissue. Also, RSV inhibiting the *NF-κB* factor mitigates the overall inflammatory status of TNF-α-induced (1, 5, and 20 μM of RSV in EA.hy926 endothelial cells) [40]. Also, prostaglandin E_2_ (PGE_2_) is actively involved in vascular inflammation, and it is synthesized by *COX-2*. RSV inhibits *COX-2* activity and expression, consequently inhibiting PGE_2_ production [50]. Moreover, using RSV 50 μM in cultured cortical cells improves atherosclerosis disease by blocking IL-6 gene expression and, in turn, IL-6 synthesis; IL-6 is a circulating cytokine recognized as another inflammatory marker found in atherosclerotic plaques [51].

The use of RSV 100 μM in human macrophages also inhibits the release of interleukin-8 (IL-8) and granulocyte macrophage colony-stimulating factor, which is involved in the recruitment of inflammatory leukocytes [52].

The vascular damage that occurs in atherosclerosis is also due to damaged mitochondrial biogenesis, which results in ROS generation and oxidative processes [53]. The use of RSV at 1–10 μM in human coronary artery endothelial cells (HCAECs) was shown to be associated with enhanced mitochondrial biogenesis and an increased protein expression of some mediators involved in the electron transport chain, suggesting the antioxidant activity of RSV [54]. Moreover, RSV triggering a direct ROS scavenging can enhance NO production or cellular-enzymatic protection by targeting the *Akt*/*eNOS* axis. In addition, regarding the antioxidant protection, in cultured human endothelial cells and in cardiac tissue of ApoE^−/−^-KO mice as well, RSV (10–100 μM) enhances the activity of some antioxidant enzymes like heme oxygenase-1 (*HO-1*), *SOD*, *CAT*, and *GPx* [55].

So, as we can see, there are several mechanisms explaining the positive role of RSV in containing the pathophysiology of atherosclerosis.

#### 3.2.2. Quercetin

Quercetin (QRC) (3,3′,4′,5,7-pentahydroxyflavone) is one of the main flavanols present in our diet. It is generally found in the form of monomeric glycosides in different fruits, vegetables, and cereals, like apples, berries, red onions, grapes, broccoli, bell peppers, coriander, citrus fruits, and tea leaves (*Camellia sinensis*) [56]. Unfortunately, it has very low bioavailability because of its wide metabolism and its poor solubility [57]. After dietary intake, plasma concentrations are typically in the range of 0.3–1.5 μM, mostly represented by quercetin glucuronides and sulfates, while free aglycone is rarely found. Its metabolites are primarily produced by enterocytes and hepatocytes, and then they accumulate in the tissues shortly after the ingestion of foods containing QRC [58]. Supplementation with 500–1000 mg/day of quercetin aglycone can produce plasma levels up to 5 μM, a concentration that is still lower than the one used in in vitro studies (10–50 μM). However, conjugated metabolites can still be bioactive, and intestinal deconjugation may release active aglycone locally [57].

QRC is an indirect *SIRT1* activator; specifically, it increases the NAD^+^/NADH ratio and increases *SIRT1* mRNA. This activation promotes the deacetylation of *NF-κB*, leading overall to a reduced inflammatory gene expression; promotes the suppression of endoplasmic reticulum stress; and consequently, reduces oxidative stress damage in cardiomyocytes (QRC 25 mg/kg, 50 mg/kg, and 100 mg/kg in primary myocardial cells of suckling mice culture) [59]. *SIRT1* activation also promotes mitochondrial function and autophagy, resulting in improved vascular homeostasis and plaque stability. These findings were observed in a study where MRAW264.7 macrophage foam cells were treated with 25 and 50 μM of QRC [60].

The main feature of QRC is the antioxidant activity. QRC has a structure that includes the presence of reactive hydroxyl groups that contribute to its antioxidant activity; in fact, it inhibits nicotinamide adenine dinucleotide phosphate (NADPH) oxidases (*NOX*) and xanthine oxidases (*XO)* with a consequent reduction in ROS generation, as seen in a study using 25, 50, and 100 mg/kg of QRC in male ApoE^−/−^ KO mice. Moreover, it promotes the activation of the *Nrf2* pathway, resulting in an upregulation of the antioxidant enzymes *SOD*, *CAT*, and *GPx*, studied in high-fat diet–induced atherosclerosis in the carotid artery of rats, where a dose of 30 mg/kg/day of QRC was used. Given this antioxidant activity, QRC can reduce the endothelium damage and can reduce the formation of oxLDL, which directly contributes to the formation of foam cells (2.5, 5, 10 μM of QRC in endothelial cells) [61].

It also reduces the activity of COX and lipoxygenases (*LOX*), thus modulating the inflammatory cascade [62].

Moreover, the antioxidant property of QRC in atherosclerosis is correlated to the myeloperoxidase (*MPO*)/H_2_O_2_ system. As seen in ApoE^−/−^ mice, modulating this pathway, QRC reduces the hypochlorous acid (HOCl) levels, which play a toxic role in vascular cells, contributing to atherosclerosis [63].

Given the above-mentioned activities, it has emerged that QRC may have antiatherogenic properties, since in animal models it was shown that it can reduce atherosclerotic plaques (using 25, 50, and 100 mg/kg of QRC in male ApoE^−/−^ KO mice) [64,65]. There are different molecular mechanisms that explain the effect of QRC in atherosclerotic lesions, but the most important seems to involve the nucleotide-binding oligomerization domain-like receptor protein 3 (NLRP3) inflammasome. In fact, an overaccumulation of oxLDL causes hyperinflammation in macrophages that, in turn, activates the NLRP3 inflammasome, leading to a progressive worsening of the disease. During the inflammatory status of macrophages, there is an activation of another pro-inflammatory signaling pathway, the galectin-3 NLR family pyrin domain containing 3 (Gal-3-NLRP3); this pathway also is suppressed by QRC administration, specifically this is observed in male ApoE^−/−^ KO mice treated with 100 mg/kg QRC, thus ameliorating atherosclerotic lesions [66].

Supporting the anti-inflammatory properties of QRC, it was noticed that it can reduce the gene expression of NF-κB, resulting in a regulation of the pro-inflammatory cytokines like IL-6, IL-1β, TNF-α, IL-10, and nuclear factor of kappa light polypeptide gene enhancer in B-cells inhibitor alpha (*IκBα*). These effects were observed in a study conducted on 85 patients with coronary artery disease treated with 120 mg of QRC [62]. Given the attenuation of the inflammatory processes, there is a mitigation of monocyte recruitment and vascular inflammation. Also, in ApoE^−/−^ mice and C57BL/6J (C57) mice treated with 20 mg/kg/day of QRC, it is observed that this compound may inhibit the expression of ICAM-1 and VCAM-1 adhesion molecules, which promote the interaction between the endothelium and the leukocytes [65].

Another condition involved in the progression of atherosclerosis is the unbalanced cholesterol homeostasis and unbalanced lipid metabolism. In this regard, QRC reduces TC and TG levels and promotes the expression of *PPARγ*, calmodulin-liver X receptor α (LXRα), and ATP-binding cassette transporter A1 (ABCA1), which are some essential proteins that, increasing the macrophage reverse cholesterol transport (RCT), contribute to the cholesterol balance [67]. Moreover, QRC inhibiting the oxidation of LDL particles prevents the generation of foam cells and then the atherosclerotic plaque formation. In fact, QRC (10, 20, 40, or 60 µM) treatment improved cell viability of ox-LDL-induced RAW264.7 cells and blocked lipid accumulation [68].

Among the beneficial effects of QRC, there is the improvement of damaged endothelium. Specifically, it exerts an interesting dual role: in a condition of nitrosative stress caused by NO overproduction, QRC reduces *eNOS* activity and decreases intracellular calcium concentration, leading to endothelial cell protection [69]; on the contrary, when there is a lack of endogenous NO, QRC enhances *eNOS* activity, leading to an increased NO bioavailability, resulting in vasodilatation and reduced vascular stiffness [70].

Platelet aggregation also is inhibited by QRC. This is explained by mainly two mechanisms: the reduced synthesis of thromboxane A_2_ (TXA_2_) and the modulation of two intracellular signaling pathways, mitogen-activated protein kinase (*MAPK*) and *PI3K*/*Akt*, as seen in male C57BL/6 mice treated with 50 and 100 mg/kg of QRC. As a result, there is a reduction in atherothrombotic events, which mitigate the disease [71,72].

#### 3.2.3. Naringenin

Naringenin (NAR) (5,7-dihydroxy-2-(4-hydroxyphenyl) chroman-4 one) is a flavanone especially found in citrus fruits like grapefruit (*Citrus paradisi* L.), lemons, and oranges, where, among the beneficial effects, is responsible for the characteristic bitter aftertaste [73]. NAR shows modest oral bioavailability due to rapid metabolism; in fact, after ingestion of its dietary sources, plasma levels of free NAR usually peak at 0.5–1.5 μM within 2–4 h. The plasma concentration of NAR is generally represented by the glucuronide and sulfate conjugates. Supplementation with 500 mg–1 g/day can increase total plasma concentrations to 3–6 μM, although effective in vitro concentrations for *SIRT1* activation and antiatherogenic effects are greater than 10 μM. This discrepancy suggests that systemic effects in vivo may be mediated partly by conjugated metabolites and by local activity in the gut and vascular endothelium [73]. NAR has long been known for its antioxidant, anti-inflammatory, cardioprotective, antidiabetic, anticancer, and antiviral properties, and thanks to its low toxicity profile, it is widely used in the diet and in traditional medicine [74].

**Figure 3:**
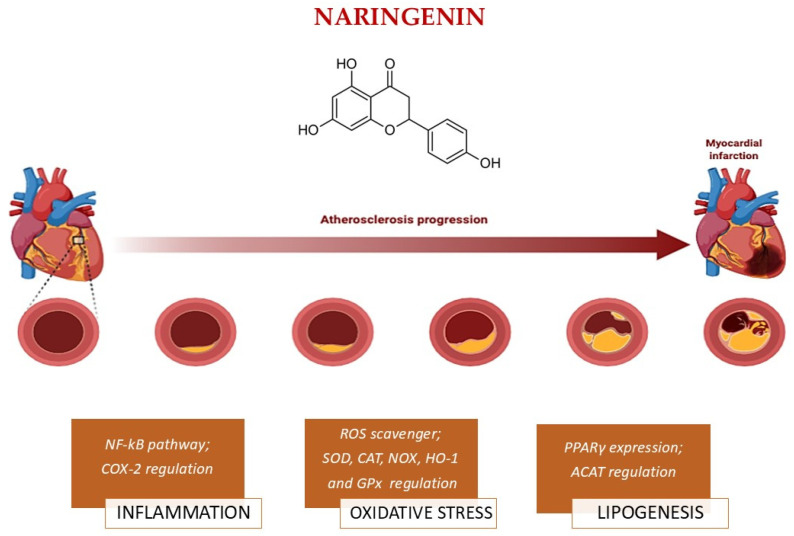
Naringenin slows down the progression of atherosclerosis through different mechanisms of action that mainly act on inflammation, oxidative stress, and lipid metabolism.

The protective role of NAR in atherosclerosis involves different mechanisms of action, and regarding its antioxidant effect, NAR acts as a strong scavenger of ROS, thereby reducing metabolic damage to lipids, proteins, and DNA (Figure 3). This effect is mainly due to the role of NAR in upregulating the expression and the activity of endogenous antioxidant enzymes like *SOD*, *CAT*, and *GPx* [75]. Additionally, NAR inhibits *NOX*, which represents an important source of ROS production in endothelial cells and macrophages, thus reducing oxidative stress [76]. Also, NAR indirectly activates *SIRT1* through *AMPK* activation and increasing NAD^+^ levels, leading to decreased ROS levels. Also, it repairs the mitochondrial electrons, transport chain and promotes *Nrf2* transcription, consequently performing an antioxidant effect [77].

The reduced production of ROS limits the oxidative modification of LDL, which, as we already mentioned, is a key step in foam cell formation and plaque development. Also, by preventing oxidative damage to the endothelium, NAR preserves NO bioavailability, leading to improved vasorelaxation, as it was demonstrated in HUVEC treated with NAR 5 to 50 μM [75]. This vasorelaxation seems to be due to the potential of NAR in promoting the opening of a calcium-activated potassium channel (BKCa), which is situated on the sarcolemmatic membrane of smooth muscle cells [78].

NAR plays an important anti-inflammatory role, targeting both endothelial cells and macrophages. In the vascular endothelium, NAR blocks the stimulation of the *NF-κB* pathway; specifically, it inhibits the phosphorylation and the nuclear transportation of the *NF-κB* p65 subunit, leading to reduced gene transcription of VCAM-1, ICAM-1, and E-selectin, as was observed in cholesterol-fed rabbits that had a daily intake of 500 mg/kg of NAR. Also, it promotes the reduction in cytokines like IL-6 and TNF-α [76,79]. In addition, NAR mediates *Nrf2* activation and *HO-1* mRNA expression, as seen in H9c2 cells treated with 40 μg/mL of NAR [80].

NAR exerts its anti-inflammatory properties also by decreasing the production of the pro-inflammatory eicosanoid, PGE_2_, and reducing *COX-2* expression, as seen in the macrophage cell line J774A.1, which was treated with NAR 0.5–5–50 μM [81]. Another crucial inflammatory target is represented by matrix metallopeptidases. In fact, MMP9 expression seems to be reduced by NAR under 10–25 μM concentration through the reduction in VSMC migration [82]. As a result, there is a reduced monocyte adhesion, a crucial step in plaque initiation.

NAR also exerts its role in macrophage lines at concentrations between 5 and 50 μM, where it exhibits a strong potential to reduce the production of IL-6, IL-8, IL-1β, and TNF-α [83].

Regarding lipid metabolism, NAR decreases LDL-C and TG levels while increasing HDL-C, both in an in vivo animal model of high-fat-diet-fed SD rats fed with NAR (100, 200, 400 mg/kg) and in an in vitro model of 3T3-L1 adipocyte cells treated with NAR 25–75 μg/mL [84]. Also, it emerged that it could decrease the hepatic cholesterol acyltransferase (*ACAT*) activity, leading to reduced atherosclerotic plaque progression [85].

NAR has been shown to inhibit the hepatic synthesis and secretion of apolipoproteinB (ApoB)-containing lipoproteins, in particular very low-density lipoprotein, through post-endoplasmic reticulum presecretory proteolysis mechanisms. These events result in lower circulating levels of LDL and VLDL. Moreover, NAR enhances the expression of PPARα, which promotes fatty acid oxidation and reduces hepatic lipid accumulation [75].

#### 3.2.4. Curcumin

Curcumin (CRC) ((1E,6E)-1,7-bis (4-hydroxy 3-methoxyphenyl)-1,6-heptadiene-3,5-dione) is a natural polyphenol and a natural yellow pigment derived from turmeric (*Curcuma longa* L.). This plant is commonly used, especially in South Asia, as a dietary spice, but also in traditional medicine due to its several beneficial effects [86].

CRC has been widely studied due to its anti-inflammatory, antioxidant, cardioprotective, anti-diabetes, anti-aging, and anticancer properties [87,88].

It is demonstrated that an intake of up to 8 g per day is safe; in fact, it has very low oral bioavailability, principally caused by its low solubility in water, fast metabolism, and low intestinal absorption [89]. After oral doses of 2–8 g/day in humans, plasma concentrations are usually undetectable or in the low nanomolar range, under 50 μM, with most circulating as glucuronide and sulfate conjugates. To overcome these problems, different strategies have been applied, aimed at improving its pharmacokinetic profile. In fact, alternative formulations have been prepared, such as the following: nanoformulations, phospholipid complexes, and co-administration with piperine, a plant alkaloid found in Piper nigrum Linn. Piperine is generally used in combination with CRC thanks to its potential in reducing the rate of intestinal and hepatic metabolism [90]. Formulations with 2 g of curcumin and 20 mg of piperine can increase bioavailability by 200%. However, even with enhanced formulation, plasma levels typically reach 0.2–2 μM, which is still lower compared to the range of 5–20 μM that is generally used in in vitro studies [88].

CRC is a *SIRT1* indirect activator since it promotes *SIRT1* transcription and mitigates oxidative stress and inflammation, thus exerting protection in the vascular wall. In vitro studies on endothelial cells report activity at low nanomolar or micromolar concentrations (0.1–20 μM). Regarding *SIRT1* activation, it has been demonstrated that CRC at doses between 20 and 50 mg/kg also promotes the stimulation of the *SIRT1*/*Nrf2* pathway and inhibits Toll-like receptor 4 (TLR4) expression in newborn rats. Furthermore, it emerged that also a CRC metabolite, tetrahydrocurcumin, upregulates the expression of *SIRT1* and deacetylates the enzyme *SOD*2, as shown in vitro and in vivo studies as well [91].

Regarding its antioxidant effect, CRC activates the *Nrf2*/ARE signaling pathway, resulting in the upregulation of the antioxidants *SOD*, *HO-1*, *GPx*, and *CAT*. These effects lead to a reduction in ROS and to an inhibition of LDL oxidation, thus protecting vascular cells from oxidative injury [92].

CRC also is involved in the mitigation of vascular inflammation since it suppresses inflammation, mainly inhibiting the *NF-κB* pathway. This leads to a downregulation of TNF-α, IL-1β, and IL-6. It also reduces monocyte adhesion to the endothelium since CRC inhibits ICAM-1 and VCAM-1 expression. These mechanisms were observed in human endothelial cells and monocytes, where it has been found that 2 μg/mL of CRC reduces IL-1β in HUVECs and reduces IL-6 and TNF-α in THP-1 cells [93].

Regarding the implication of CRC in modulating the lipid metabolism, it has been observed that the in vitro treatment of endothelial cells with CRC 0.5, 1, and 2 μM, leads to reduced lipid deposition, so reduced formation of atherosclerotic lesions. Also, in vivo, in ApoE^−/−^ mice, it has been shown that using 25 mg/kg/day of CRC can decrease LDL-C, TC, and TG while increasing HDL-C levels, resulting in a significantly decreased formation of atherosclerotic plaque [94].

It also influences lipid homeostasis in cells since it promotes the downregulation of some scavenger receptors like CD36 and scavenger receptor class A (SR-A1), which in turn promotes the uptake of oxLDL in macrophages. Moreover, CRC significantly promoted cholesterol efflux in a dose-dependent manner (10, 20, and 40 µM) in macrophages, upregulating the transporters ABCA1 and LXRα and resulting in reduced foam cell formation. Overall, these mechanisms suggest the role of CRC in the atherosclerotic plaque regression [95].

#### 3.2.5. Berberine

Berberine (BBR) (9,10-Dimethoxy-7,8,13,13a-tetradehydro-2′H-[1,3] dioxolo [4′,5′:2,3] berbin-7-iumis) is a natural isoquinoline generally used in China like a traditional medicine. It is extracted from different plants, especially from *Berberis vulgaris* (barberry), *Hydrastis canadensis* (goldenseal), and *Coptis chinensis* (Chinese goldthread) [96].

BRB shows a very low oral bioavailability due to its wide metabolism and poor intestinal absorption. After oral doses of 500 mg–1.5 g/day, plasma concentrations of free berberine are usually in the low nanomolar range (<0.1 μM). However, multiple active metabolites (mainly berberrubine, thalifendine, and demethylenberberine) reach higher systemic levels and contribute significantly to pharmacological effects. Despite low systemic concentrations, BRB accumulates in tissues such as the liver, intestine, and vascular wall, where it can exert its actions. In vitro studies often use 1–50 μM concentrations, highlighting a gap compared to circulating levels [96].

Among its beneficial effects, particularly interesting is its cardioprotective effect, especially in atherosclerosis. In fact, as seen in ApoE^−/−^ mice treated with BRB dissolved in drinking water (0.5 g/L), there is an interesting reduction in tissue inflammation and atherosclerosis. A key mechanism is represented by the upregulation of *SIRT1* expression, which in turn leads to an increased deacetylase activity that promotes the deacetylation of NF-κB, p65, FOXO1, and *PGC-1α*, resulting in reduced inflammation, oxidation, and improved mitochondrial function [97]. In fact, BBR provides endothelial protection in ApoE^−/−^ mice treated with 78 and 156 mg/kg of BRB through its implication in mitochondrial dysfunction and fatty acid β-oxidation [98].

Moreover, indirectly activating *SIRT1* through the NAD^+^ synthesis pathway, the mitochondrial modulation, and the *AMPK* activation, BBR promotes the nuclear migration and deacetylation of the transcription factor EB (*TFEB*), resulting in the autophagy of peritoneal macrophages [99]. Also, BBR improves *SIRT1* upregulation in the aging heart, where there is a deficiency of Klotho (KL), an anti-aging protein. Low KL levels seem to be correlated with atherosclerosis, and BBR upregulating *SIRT1* also increases KL expression, leading overall to a protective condition [100].

The vascular protection of BBR is also guaranteed by its role in upregulating *eNOS* and increasing NO bioavailability and reducing *LOX-1* expression, leading overall to vascular relaxation and reduced endothelial dysfunction [101]. Also, BBR downregulates the adhesion molecules ICAM-1 and VCAM-1 expression and limits the cell proliferation rate of HUVECs, supporting once again its protective role in vascular damage [101].

BBR exerts antioxidant effects, partly mediated by *AMPK* and *SIRT1* activation, which in turn regulate the FOXO transcription factor that is involved in oxidative stress resistance through the stimulation of *SOD* and *GPx*. In addition, it inhibits the activation of *NOX*, thus resulting in reduced ROS levels [97].

Like the other *SIRT1* activators, BBR shows anti-inflammatory effects by regulating *NF-κB* signaling inhibition, then reducing TNF-α, IL-1β, and IL-6 levels and increasing IL-10 and adiponectin levels, as seen in in vivo animal models, male ApoE^−/−^ mice, and C57BL/6J mice treated with 50 mg/kg/daily [102].

The beneficial effect of BBR in atherosclerosis is explained also by its implication in lipid metabolism. In fact, it shows anti-hypercholesterolemic effects, decreasing the levels of TC, TG, LDL-C, and improving HDL-C levels, as was observed in Caucasian subjects treated with 500 mg of BRB twice a day [103]. Also, it promotes the leptin-to-adiponectin ratio in patients with increased cardiovascular risk [104]. Some in vivo animal studies, which used male Sprague–Dawley rats, indicate that the treatment with 50, 100, and 150 mg/kg/daily of BBR can decrease blood cholesterol levels derived by an inhibited intestinal absorption and can decrease cholesterol uptake by enterocytes [105].

Recently, a correlation has been found between trimethylamine (TMA)/trimethylamine N-oxide (TMAO) production in the gut microbiota and its implication in the development of on atherosclerotic lesion. In fact, after a single oral dose of BBR (100 mg/kg), TMA and TMAO levels in high fat diet-fed hamsters significantly decreased [106].

#### 3.2.6. Fisetin

Fisetin (FIS) (2-(3,4-Dihydroxyphenyl)-3,7-dihydroxy-4H-1-benzopyran-4-one) is a flavonoid (specifically a flavonol) present in many plants, where it acts as a yellow/ochre dye. Among edible plants, FIS is found in different fruits and vegetables, especially in strawberries, apples, persimmons, onions, and cucumbers. It is also found in nuts and wine [107]. Despite promising biological activity in preclinical studies, its oral bioavailability in humans remains poorly characterized. Animal studies show rapid metabolism with extensive phase II conjugation (glucuronidation and sulfation), resulting in low circulating aglycone levels. The higher FIS concentration (2.53 g/mL) was reached after 15 min of i.p. treatment (223 mg/kg), therefore leading to the need for repeated administrations [108]. In a first-in-human study, using a novel formulation of an encapsulated form of hydrogel fisetin, the bioavailability and pharmacokinetics are significantly enhanced, yielding good results using doses of about 200 mg of FIS, compared to the 1000 mg previously used to reach the same plasma peak. Also, plasma level was quantifiable up to 8 h after FIS consumption, suggesting that this formulation provided protection of the bioactive molecule [107]. Improved formulations (liposomal, nanoparticle-based) are under development to enhance FIS bioavailability and translational relevance.

Like other polyphenols and flavonoids, FIS has significant medicinal potential and many beneficial effects for human health. In particular, it is considered a senotherapeutic agent, which means that it can kill senescent cells, promoting cellular renewal prolonging lifespan [108]. In fact, it has been demonstrated in male ApoE^−/−^ mice that 12.5 mg/kg of FIS can reduce the expression of some aging mediators like p21, p53, and p16 [109].

Emergent studies also suggest that FIS and its derivatives exert anti-atherosclerotic properties [110].

As a demonstration, it has been observed that it has antioxidant effects. There are different mechanisms underlying this activity: first, FIS enhances the activity of antioxidant enzymes such as *SOD*, *CAT*, and *GPx*, as was observed in a human study of healthy subjects treated with 1000 mg of FIS [107]; moreover, it plays a cytoprotective role against oxidative stress by regulating protein kinase C-delta (*PKC-δ*), p38, and *Nrf2*-ARE signaling pathways, which consequently are involved in the upregulation of *HO-1* expression in human pulmonary artery endothelial cells (HPAECs) after the use of 10–200 μg/kg [111]. Also, it inhibits the activation of *NOX*1, resulting in a reduction in ROS production [110]. By reducing oxidative stress, FIS 20 μM prevents the oxidation of LDL, a crucial event in foam cell generation and early plaque development in U937-derived macrophages. Additionally, it prevents atherosclerotic lesions by inhibiting copper ion-dependent LDL oxidation, consequently blocking the linking between the ox-LDL and the Class-B scavenger receptor CD36 on macrophages [112].

Regarding its anti-inflammatory effects, FIS plays a central role both in endothelial cells and macrophages. In endothelial cells, FIS exhibits anti-inflammatory properties; in fact, one of the mechanisms proposed involves the High Mobility Group Box 1 (HMGB1) signaling pathway. The inhibition of this pathway leads to a suppressed TNF-α production and to a suppressed activation of some pro-inflammatory mediators such as *Akt*, *NF-κB*, and Erk½ [113].

It emerged that the anti-inflammatory properties of 30 µM FIS are also explained by its role in inhibiting *COX-2* and *MMP-9* in human brain microvascular endothelial cells (HBMEC) [114].

On the other hand, in macrophages, the treatment with 10, 30, and 100 μM of FIS inhibits the expression of some pro-inflammatory genes such as monocyte chemoattractant protein-1 (MCP-1), IL-1β, *iNOS*, urokinase plasminogen activator (uPA), and its receptor uPAR [115].

Also, the use of FIS 5, 10, and 20 μM attenuates NO and IL-6 production and the phosphorylation of *MAPK* and MMP in mouse macrophage cell line RAW264.7 [116]. Moreover, FIS suppresses TNF-α production by the downregulation of Src-family or Syk tyrosine kinases and the nuclear translocation of p65/*NF-κB,* as demonstrated by the use of 5–160 μM of FIS in mouse macrophage cell line RAW264.7 [117].

As a promising antiatherosclerotic agent, FIS is also involved in the regulation of lipid metabolism, where it is observed that the use of 12.5 mg/kg of FIS in male apoE^−/−^ mice can lower TC, LDL-C, and VLDL-C levels [109].

FIS exerts antiplatelet effects by inhibiting platelet aggregation along with ATP release [118]. Also, it inhibits PKC activity, an essential enzyme involved in platelet regulation, leading to an inhibition of thrombin activity, which is actually involved in the morphology alterations of platelets [119].

FIS is known to be a *SIRT1* activator. In fact, it promotes a weak/modest direct activation and a strong indirect activation by increasing *SIRT1* expression and activating the *AMPK* pathway. Overall, it contributes to the deacetylation and inhibition of NF-κB, thereby reducing the expression of pro-inflammatory cytokines [117]. Additionally, FIS enhances endothelial function through *SIRT1*-mediated deacetylation of *eNOS*, leading to increased NO levels and consequently to improved vascular relaxation [120].

#### 3.2.7. Piceatannol

Piceatannol (PCT) (trans-3,4,3′,5′-tetrahydroxystilbene or 3,3′ 4,5′-tetrahydroxy-trans-stilbene) is a natural compound found in different plants and foods like sugarcane, berries, passion fruit seeds, peanuts, grapes, wine, and white tea [121]. Due to its similar structure to RSV, it was hypothesized that PCT may also prevent or improve atherosclerosis. In addition to natural sources, PCT is also a product of RSV metabolism; however, its oral intake is followed by the formation of some metabolites that derive from phase II metabolism; on the contrary, phase I enzymes have no significant effects on its metabolism. The main transformations are glucuronidation, methylation, and sulfation, resulting in different metabolites like rhapontigenin, isorhapontigenin, and mono- and di-glucuronide piceatannol [121]. Compared to RSV, seems to show a better bioavailability profile; however, there are just a limited number of works proving this.

In fact, it exerts antioxidant effects, since it was shown that 30 mg/kg/day of PCT intravenous injection inhibits arginase activity in male wild-type mice. When this enzyme is blocked, there is a restoration of L-arginine availability, which in turn promotes *eNOS* dimer stabilization, leading to increased NO production and reduced oxidative stress [122].

PCT has a scavenger activity since it reduces ROS generation and protects against oxidative cell damage, and this was observed in HUVEC, where PCT (100 µM) treatment significantly lowered palmitic acid-induced ROS and suppressed *NF-κB* activation, demonstrating its potential as an antioxidant and anti-inflammatory agent [123].

Regarding the anti-inflammatory effect, PCT promotes the inactivation of *NF-κB*, suppressing *IkBa* kinase activity and reducing p95 phosphorylation, leading overall to reduced pro-inflammatory gene expression of *COX-2*, *MMP-9*, *TNFα*, and IL-6 both in HUVEC and macrophages [123,124].

In addition, PCT promotes the activation of *Nrf2*, a key regulator of antioxidant enzymes, suggesting a boosting defense through *HO-1* expression. The pivotal modulation of the *Nrf2* and *NF-κB* pathways explains the multitarget activity of this polyphenolic compound, which suppresses both oxidative stress and inflammatory processes, mitigating endothelial dysfunction, foam cell generation, and atherosclerotic plaque progression [125].

The endothelial function is also regulated by PCT through the inhibition of the proliferation and migration of VSMCs. The main mechanism underlying this event involved the suppression of several pathways like Erk½, c-Jun N-terminal kinases (*JNK*), *NF-κB*, and *PI3K*/*Akt*, consequently leading overall to a reduced MMP9 expression. These events overall prevent vascular remodeling, as seen in VSMCs using different concentrations of PCT (25–300 μM) [126].

Additionally, PCT modulates lipid metabolism through multiple pathways, counteracting once again atherosclerosis progression. In fact, from a study made in HepG2 human hepatoma cells using PCT 100 μM, it emerged that this bioactive molecule can inhibit lipogenesis by blocking key transcription factors and enzymes like sterol regulatory element-binding transcription factor 1 (*SREBP-1*), acetyl-CoA carboxylase (*ACC*), and *PPARγ*, leading to reduced TG accumulation [127]. Also, it downregulates the fatty acid transporter CD36, reducing lipid uptake, and reduces lipolysis by inducing autophagy-dependent degradation of adipose triglyceride lipase (*ATGL*), comparative gene identification-58 (CGI-58), and perilipin 1 (PLIN1). Overall, these mechanisms lead to reduced circulating free fatty acids [128]. These findings were reached both in vitro, using PCT concentrations (0–50 µM) in 3T3-L1 preadipocytes, and in vivo in five-week-old male C57BL/6 mice treated with 10 mg/kg intraperitoneal injection of PCT [128].

These findings hypothesize the protective role of PCT in atherosclerosis, which often is related to the direct activation of *SIRT1,* since it has an analog structure of RSV that binds the allosteric site, and to the indirect activation promoted by the antioxidant and anti-inflammatory actions [129].

#### 3.2.8. Honokiol

Honokiol (HNK) (3′,5 di 2 propenyl 1,1′ biphenyl 2,4′ diol) is a bioactive neolignane compound extracted from Magnolia species. HNK oral bioavailability is relatively low, mainly due to its hydrophobic nature and large first-pass metabolism in the liver; in fact, in rodent models, oral administration has demonstrated a bioavailability of about 5%. The metabolism involves cytochrome P450, which is implicated in the formation of the 4-O-methylhonokiol derivative, rapidly converted to HNK in a concentration-dependent manner. Studies in vitro and ex vivo in rats, using a 10µM concentration of HNK, show that its metabolism leads to the formation of two main metabolites, honokiol monoglucuronide and honokiol monosulfate, suggesting that glucuronidation and sulfation are the main transformations that contribute to HNK clearance [130]. A single oral dose of HNK (40 mg/kg b.w.) in rats is rapidly absorbed. The plasma peak is reached within 20 min then is rapidly metabolized to mono-glucuronidated HNK, which in turn is slowly eliminated with a plasma half-life of 290.4 min. To overcome the limitations of HNK bioavailability, several formulation strategies have been explored, like nanoparticles and liposomal formulations, co-delivery systems, and chemical modifications [130]. Just like the other *SIRT1* activators, exerts antioxidant, anti-inflammatory, and lipid-modulating activity.

As an antioxidant molecule, it has a scavenging activity towards ROS and reactive nitrogen species (RNS) too. Moreover, HNK inhibits Fe(III) adenosine diphosphate (ADP)/NADPH and Fe(III) ADP/NADH-induced lipid peroxidation, leading to a regulation of the mitochondrial redox homeostasis [131]. Regarding its antioxidant activity, HNK (20 mg/kg) was studied in male C57BL/6 mice, where it has been observed that it may activate the *AMPK*/*SIRT3* signaling pathway, inducing *AMPK* phosphorylation and nuclear transportation of *Nrf2*, leading overall to enhanced *SOD* and *CAT* activity and thus reducing ROS accumulation. Also, it directly inhibits lipid peroxidation [132].

In addition, enhancing the Keap1/*Nrf2*/ARE axis, HNK promotes the expression of the antioxidant glutathione (GSH), as seen in HUVECs treated with HNK 10 μM [132].

Also, the use of 2.5, 5, and 10 μM of HNK in rat aortic smooth muscle cells (RASMCs) exerts anti-inflammatory properties since it inhibits some pro-inflammatory signaling pathways such as NF-κB, *MAPK*, and *SIRT3* pathways, leading to a downregulation of the cytokines TNF α, IL-6, and IL-1β [133,134]. Moreover, HNK has been demonstrated to suppress the activation of pro-inflammatory enzymes, like *COX2, NOS*, and *iNOS*, confirming once again its anti-inflammatory power [135].

The antiatherosclerotic activity of HNK is also supported by a study where HUVECs were treated with HNK 10 μM, resulting in antithrombotic activity since it inhibits TXA_2_ formation and antiplatelet activity since it inhibits platelet aggregation by decreasing agonists like ADP and collagen [132]. Furthermore, it prevents vascular damage by promoting the release of NO, consequently leading to vascular relaxation [132]. HNK downregulates the adhesion molecules ICAM-1 and VCAM-1, enhancing endothelial improvement [135].

HNK is involved in lipid metabolism, where activating the axis SIRT3/*AMPK* triggers lipophagy and mitochondrial lipid utilization in hepatocytes, counteracting lipid-driven vascular damage in AML12 cells [136]. It has been observed that it can reduce the levels of TC, TG, and LDL-C and increase the level of HDL-C [132].

In addition, HNK 5 μM increases *SIRT1* expression and activity in HUVECs when they are subjected to stress stimuli, leading to reduced ER stress and apoptosis *SIRT1*/*Akt*-mediated, thus preventing endothelial damage [137].

#### 3.2.9. Epigallocatechin-3-Gallate

Epigallocatechin-3-gallate (EGCG) (2R,3R)- 5,7-Dihydroxy- 2- (3,4,5-trihydroxyphenyl)-3,4-dihydro-2H-1-benzopyran-3-yl 3,4,5-trihydroxybenzoate) is the main polyphenolic catechin in green tea and has been widely studied for its valuable effects in atherosclerosis via different pathways [138].

However, its clinical application is limited by low oral bioavailability, rapid metabolism, and systemic clearance. Following oral administration in humans, EGCG reaches plasma peak concentrations within 1–2 h, but most of the dose is quickly metabolized through methylation, sulfation, and glucuronidation and then excreted with the urine within 8 h [139]. Plasma bioavailability is typically low; in human studies, the common oral doses of 200–800 mg/day are often divided into multiple doses. In rodent models, doses of 10–100 mg/kg/day are frequently used to mimic the plasma concentrations achieved in humans. Strategies such as co-administration with piperine or encapsulation in nanoparticles have been explored to improve stability and systemic exposure [139].

Its antioxidant activity has been demonstrated by the potent scavenging effect against ROS and by the ability to restore the endogenous antioxidant defense. In fact, EGCG reduces NADPH oxidase-mediated ROS and enhances *SOD* activity, thus protecting endothelial cells from homocysteine-induced damage [138]. In addition, EGCG promotes the stimulation of the *Nrf2* pathway and *HO-1* activity, ultimately upregulating the cytoprotective genes transcription, thereby mitigating oxidative stress, suppressing inflammation, and pyroptosis [138]. In vitro vascular and inflammatory models, like endothelial cells and macrophages, typically use EGCG 1–50 μM.

Regarding its anti-inflammatory potential, EGCG downregulates different inflammatory mediators like TNF-α, CRP, and VCAM-1 and ICAM-1-related mRNA synthesis. This condition results in a lowered rate of erythrocyte sedimentation in a reduction in the total leukocyte and other inflammatory parameters, suggesting its important role in atherosclerosis. This anti-inflammatory activity has been observed in in vivo rodent studies, which commonly administer green tea extracts or isolated EGCG at doses ranging from 10 to 100 mg/kg/day [140,141].

Also, EGCG suppresses different inflammatory signaling pathways, like *NF-κB*, leading to a reduced production of MCP-1 factor in HUVEC cells [140]. Additionally, it inhibits the phosphorylation of p38 *MAPK* and *Erk* in the *MAPK* pathway, thus suppressing the p38 *MAPK* signaling pathway and resulting in the prevention of cell adhesion [141]. EGCG exerts its anti-inflammatory effect in macrophages as well, where at a concentration of 1 μM, it decreases the expression of TLR4, which is implicated in all the inflammatory stages of atherosclerosis [142].

Finally, EGCG interferes with the Notch signaling pathway, which plays a central role in the inflammatory response. In fact, it has been observed that in damaged blood vessels there is an increased number of notch ligand receptors, while EGCG, binding to notch receptors, inhibits the inflammatory notch cascade [143].

EGCG modulates lipid metabolism also, as it can directly reduce TG levels. This effect is mediated by the suppression of fatty acid synthase (*FAS*) activity or by the downregulation of *PPARγ* and *FAS* through the *PI3K*/*Akt* signaling pathway [144]. The effect of green tea polyphenols on gut microbial diversity and fat deposition was studied in C57BL/6J HFA mice, which were treated with EGCG 0.05%, 0.2%, and 0.8% [144].

In addition, EGCG lowers TC and LDL-C and increases HDL-C, partly through the inhibition of hepatic SREBP1 and activation of liver X receptor-mediated cholesterol efflux genes like ABCA1, ABCG5/8, and LXRα/β [145]. Another mechanism involved in the reduced hepatic cholesterol production is the axis *SIRT1*/FOXO1/SREBP-2, which leads to reduced liver lipid deposition and reduced serum TG and LDL-C levels [146].

Serving as an indirect *SIRT1* activator, EGCG counteracts the homocysteine-induced endothelial apoptosis by enhancing *SIRT1* expression and *AMPK* activity and stabilizing *eNOS*/*Akt* signaling, thus contributing to the maintenance of endothelial survival [147].

#### 3.2.10. Hydroxytyrosol

Hydroxytyrosol (HT) (3-hydroxytyrosol, 3,4-hydroxyphenyl ethanol), which is found in high concentrations in olives and virgin olive oil, is a natural phenolic compound particularly known for its antioxidant, anti-inflammatory, anti-atherogenic, and antimicrobial properties [148]. Due to a high metabolism both phases I and II, it has a poor bioavailability [149]. In fact, after ingestion, HT is rapidly absorbed, with plasma peak concentrations occurring about 30 min post-consumption. After the first-pass metabolism, conjugated metabolites such as HT sulfate and 3,4-dihydroxyphenylacetic acid are formed. Studies have shown that the bioavailability of HT is influenced by the food matrix; specifically, the consumption of extra virgin olive oil results in higher plasma concentrations compared to other matrices [150]. In human studies, oral doses of HT typically range from 5 to 30 mg/day. However, achieving therapeutic concentrations may require higher doses or the use of advanced delivery systems to enhance bioavailability [151].

HT has an ortodiphenolic structure, which is responsible for its antioxidant activity; in fact, it reduces ROS and malondialdehyde (MDA) levels and enhances NO generation in vitro and in vivo [152]. NO generation is promoted by the upregulation of the *eNOS* expression, which in turn is regulated by *SIRT1* activation. HT acts as an indirect activator of *SIRT1* since it increases *SIRT1* expression and activity through antioxidant and anti-inflammatory effects. Impaired *SIRT1* activity is related to altered vascular NO synthesis, inflammation, and vascular aging [1]. Also, HT, by targeting *SIRT1*, reduces ROS production [153]. Moreover, HT contributes to the activation of *AKT*1, leading to reduced superoxide production [154]. *SIRT1* activation HT-mediated is involved also in the save of TNF-α-induced vascular adventitial fibroblasts, leading to the maintenance of endothelial function [155].

Regarding its role in oxidative stress, HT promotes the healing of atherosclerotic plaques, increasing the nuclear accumulation of *Nrf2* and *HO-1* expression by targeting the *PI3K*/*Akt* and Erk½ signaling pathways [156]. Also, it upregulates *SOD* activity, leading to reduced mitochondrial superoxide production [157].

HT plays an important role in vascular protection; it has been observed that a supplementation of 10 mg/kg of olive extract to mice resulted in decreased E-selectin, MCP-1, and ICAM-1 expression in the endothelium [158], and 50 mg/kg of HT reduced lipid peroxidation and increased GSH expression [159]. In porcine aortic ECs also, it has been shown that HT decreases E-selectin, P-selectin, VCAM-1, and ICAM-1 expression [160]. The same results are observed in HUVEC as well [157]. HT also exerts anti-platelet effects by inhibiting TX and prostacyclin synthesis [159]. Interestingly, the HT metabolite, hydroxytyrosol-3-O-sulfate, reduces adhesion molecules like MCP-1, preventing vascular stiffness [157]. HT also exerts anti-inflammatory properties by inhibiting the expression of *COX2*, IL-1β, IL-6, and TNF-α through the *SIRT6/PKM2* signaling pathway [152].

Regarding lipid metabolism, HT regulates the reverse cholesterol transport pathway by increasing ABCA1 expression and activating the *AMPK* pathway and phosphorylation of p38 [161].

For all the above-mentioned nutraceuticals, there are just a few human studies, so we summarize them in a table in order to have a clearer overview (Table 2).

### 3.3. Safety Considerations

While several preclinical and clinical studies support vascular and metabolic benefits for the reviewed compounds, safety, drug–nutrient interactions, and dose-dependent adverse effects must be acknowledged. First of all, generalizing to all the nutraceuticals, we can affirm that supplements differ from each other in terms of purity, dosage, and formulation, so adverse events are more frequently associated with concentrated extracts compared to dietary intake; polypharmacy and underlying liver/kidney disease increase the risk of clinically relevant interactions or toxicity; many studies use concentrations well above typical free plasma levels, so toxicity at high experimental doses does not necessarily predict clinical harm, but high-dose supplementation can create risks not present with dietary exposure. Below we summarize the main safety considerations for each compound and practical recommendations for interpretation and clinical translation.

#### 3.3.1. Resveratrol

It usually is well tolerated in short-term trials but may cause gastrointestinal symptoms (nausea, diarrhea) and headache at high doses; there are reports of interactions with anticoagulants/antiplatelet agents and possible enhancement of anticoagulant effects; caution is advisable in patients on warfarin or other drugs with narrow therapeutic indices [178].

#### 3.3.2. Quercetin

Human intervention studies report few and generally mild adverse events at typical supplement doses (≤1 g/day), but long-term safety data at high doses are limited. There exist some potential interactions via phase II metabolism, but they are currently considered modest; monitor when co-administered with drugs that undergo extensive glucuronidation/sulfation [57].

#### 3.3.3. Naringenin

NAR is a component of grapefruit responsible for clinically important interactions with many orally administered drugs through inhibition of intestinal CYP3A4 and transporters (OATP), potentially increasing plasma exposure of drugs (e.g., certain statins, calcium-channel blockers, and immunosuppressants). Caution is recommended with concentrated supplements or high intake of grapefruit products in patients on CYP3A4/OATP substrate drugs [179].

#### 3.3.4. Curcumin

Generally safe at culinary intake levels, but some case reports indicate rare instances of drug-induced liver injury (DILI) associated with turmeric/curcumin supplements: risks appear greater with high-dose, long-term use and with bioavailability enhancers such as piperine. Curcumin may also interact with anticoagulants and some CYP enzymes; caution in patients with liver disease or taking hepatically metabolized drugs [180].

#### 3.3.5. Berberine

BRB is generally tolerated for metabolic indications, but it has a potential drug–drug interaction: inhibitory effects on P-glycoprotein and several CYP isoforms (e.g., CYP2D6, CYP2C9) and variable effects on CYP3A4. BRB can also lower heart rate and blood pressure in sensitive patients and may interact with other hypoglycemic or cholesterol-lowering agents; monitor when combined with prescription drugs [181].

#### 3.3.6. Fisetin

Preclinical safety is encouraging, and human safety data are emerging; early human studies report few adverse events, but long-term safety and high-dose effects are not yet fully characterized. Exercise caution in the absence of extensive clinical safety data, particularly in vulnerable populations (pregnancy, severe hepatic/renal impairment) [182].

#### 3.3.7. Piceatannol

Human pharmacokinetics (PK) and safety data are limited. Preclinical toxicology indicates dose-dependent cytotoxicity in some models; available human studies (e.g., passion fruit seed extract) report modest tolerability, but the systemic exposure is generally low. Until larger human safety datasets are available, avoid high-dose supplementation and monitor for unexpected adverse events [183].

#### 3.3.8. Honokiol

Animal studies and toxicology reviews show relatively low acute toxicity, but human PK/safety data are rare. Potential central nervous system (CNS) effects (sedation) and interactions with CNS-active drugs have been reported; standardized extract dosing and close monitoring are recommended when used with sedatives or other CNS-active medications [130].

#### 3.3.9. Epigallocatechin-3-Gallate

Green tea consumption is generally safe; concentrated green tea extracts (GTE/EGCG supplements) have been linked to cases of acute liver injury, some severe. Hepatotoxicity risk increases with high-dose supplements. EGCG also has antioxidant/pro-oxidant dose-dependent activity and may interact with certain drugs (e.g., reducing the bioavailability of some medications or affecting drug metabolism). Caution with high-dose supplements, especially in individuals with liver disease or who use hepatotoxic drugs [184].

#### 3.3.10. Hydroxytyrosol

Hydroxytyrosol has a good safety profile in animal and human studies; regulatory evaluations have concluded safety use levels for novel food applications. Nevertheless, as with other polyphenols, concentrated extracts at pharmacological doses require monitoring for potential interactions and idiosyncratic events [185].

## 4. Discussion

Atherosclerosis is a multifactorial and progressive pathology that underlies most cardiovascular diseases. Despite advances in pharmacological treatments, the cardiovascular risk is high, and there is an increasing interest in complementary strategies targeting the early mechanisms of the disease. Among these, the activation of *SIRT1* has emerged as a promising therapeutic target. *SIRT1* is a key regulator of vascular homeostasis, exerting protective effects against oxidative stress, inflammation, endothelial dysfunction, lipid accumulation, and defective autophagy, which are consistent hallmarks of atherosclerosis. A growing number of natural bioactive compounds, derived from dietary sources or traditional medicinal plants, like resveratrol, quercetin, naringenin, curcumin, and others, have been shown to act through *SIRT1*-dependent and *SIRT1*-independent pathways and mitigate atherosclerotic processes. In this review, we would like to emphasize the importance of these polyphenols, flavonoids, alkaloids, and terpenoids that could represent an interesting dietary intervention. The use of these compounds may be considered as a multi-target approach that combines nutraceuticals with lifestyle interventions or pharmacological therapies and may represent an innovative strategy for preventing or slowing the progression of atherosclerosis. Unlike conventional drugs that often target a single pathway, these molecules may simultaneously modulate different hallmarks with fewer side effects and better long-term tolerability. In fact, when comparing the overall findings, it is evident that these compounds share convergent mechanisms of action: activation of *SIRT1* and *AMPK*, reduction in oxidative stress and pro-inflammatory signaling, improvement of endothelial nitric oxide bioavailability, and favorable effects on lipid metabolism. Despite structural differences, these natural molecules share the same overlapping molecular pathways, suggesting that their effects may be complementary and potentially synergistic when consumed as part of a balanced diet or combined nutraceutical formulations. The selected molecules in this review are resveratrol, quercetin, naringenin, curcumin, berberine, fisetin, piceatannol, honokiol, epigallocatechin-3-gallate, and hydroxytyrosol. It has been observed that regarding oxidative stress, the main actions of these molecules are the reduction in ROS, the promotion of antioxidant enzymes such as *SOD*, *GPx*, *CAT*, and *HO-1*, and the inhibition of oxidative enzymes such as *NOX*. These compounds also exert anti-inflammatory effects, largely via NF-κB inhibition and suppression of cytokine production. Moreover, they reduce the expression of some adhesion molecules (ICAM-1, VCAM-1) and inhibit platelet aggregation, leading to endothelial protection.

However, it is not clear if the potential effects of these natural molecules are linked to the activation of *SIRT1*, since they exhibit pleiotropic mechanisms of action, all contributing to vascular homeostasis, maintenance of endothelial integrity, and reduced atherosclerotic plaque progression. So, despite encouraging these supportive findings about the potential role of natural *SIRT1* activators as complementary strategies in the prevention and management of atherosclerosis, further studies are warranted.

A major strength of this review is the focused analysis of compounds with documented molecular mechanisms, particularly regarding *SIRT1* modulation, oxidative stress, inflammation, vascular protection, and lipid metabolism. Nevertheless, a limitation of this study is that while preclinical data are abundant, clinical evidence remains limited, heterogeneous, and often based on small trials.

Future perspectives should focus on improving the bioavailability and pharmacokinetics of these compounds, since many polyphenols undergo rapid metabolism and have limited systemic exposure. Well-defined randomized clinical trials (RCTs) are urgently needed to validate the preclinical findings and determine optimal dosages, formulations, and safety profiles.

So, in conclusion, our review can be regarded as a starting point for the knowledge of the latest studies about advances in precision nutrition that could enable the identification of patient subgroups most likely to benefit from specific natural compounds, paving the way for more personalized preventive strategies.

## Figures and Tables

**Table 1:** Summary of the natural activators of *SIRT1* and their mechanisms of action against atherosclerosis, categorized by the effect they produced.

Compound	SIRT1 Activation	Oxidative Stress	Inflammation	Vascular Protection	Lipid Metabolism
**Resveratrol**	Directly activates SIRT1 via allosteric modulation; Indirectly activates SIRT1 via AMPK activation, and NAD^+^ increase.	ROS scavenger; Limits LDL oxidation; Enhances antioxidant enzymes (SOD, GPx, CAT, HO-1).	Inhibits NF-κB; Inhibits pro-inflammatory cytokines (TNF-α, IL-6, IL-8); Inhibits PGE_2_ and COX_2_ expression.	Improves endothelial function; Increases eNOS; Reduces adhesion molecules and chemokines (CCL2, CXCL1/KC, ICAM-1, VCAM-1).	Reduces TC, TG, LDL-C; increases HDL-C; upregulates LDLR expression.
**Quercetin**	Indirect activation of SIRT1. It promotes SIRT1 activation increasing NAD^+^/NADH ratio and increasing SIRT1 mRNA.	ROS scavenger; Limits LDL oxidation; Inhibits oxidative enzymes (NOX, XO); Enhances antioxidant enzymes (SOD, CAT, GPx).	Inhibits NF-κB; Inhibits pro-inflammatory cytokines (TNF-α, IL-6, IL-1β, IL-10, lkBα); Inhibits COX and LOX.	Improves endothelial function; Increases eNOS; Reduces adhesion molecules (ICAM-1, VCAM-1); Inhibits platelet aggregation (TXA_2_ reduction, MAPK and PI3K/Akt modulation); Inhibits NLRP3 activation.	Reduces TC, TG; Enhances cholesterol efflux via ABCA1 and through the upregulation of PPARγ and LXRα.
**Naringenin**	Indirect activation of SIRT1. It promotes SIRT1 activation through AMPK activation, increased NAD^+^ levels.	ROS scavenger; Limits LDL oxidation; Inhibits oxidative enzymes (NOX); Activates Nrf2; Enhances antioxidant enzymes (SOD, CAT, GPx, HO-1).	Inhibits NF-κB; Suppresses MAPK; Inhibits pro-inflammatory cytokines (TNF-α, IL-6, IL-8, IL-1β); Reduces MMP9 expression; Inhibits PGE_2_ and COX2 expression.	Protects endothelium; Reduces adhesion molecules (ICAM-1, VCAM-1).	Reduces LDL, VLDL, LDL-C, TG; Increases HDL-C; Decreases ACAT activity; Inhibits the hepatic synthesis of (ApoB)-containing lipoproteins; Enhances PPARγ.
**Curcumin**	Indirectly activates SIRT1 via AMPK activation and upregulating SIRT1 transcription.	Activates Nrf2/ARE axis; Enhances antioxidant enzymes (SOD, CAT, GPx, HO-1); Reduces ROS and inhibits LDL oxidation.	Inhibits NF-κB; Inhibits pro-inflammatory cytokines (TNF-α, IL-6, IL-1β); Inhibits TLR4 expression.	Protects endothelium; Reduces adhesion molecules (ICAM-1, VCAM-1).	Reduces LDL-C, TC, TG; Increases HDL-C; Enhances cholesterol efflux via ABCA1 and through the upregulation of PPARγ and LXRα.
**Berberine**	Indirectly activates SIRT1 via AMPK activation, mitochondrial modulation and NAD^+^ enhancement.	Reduces ROS; Inhibits oxidative enzymes (NOX); Enhances antioxidant enzymes (SOD, GPx).	Inhibits NF-κB; Inhibits pro-inflammatory cytokines (TNF-α, IL-6, IL-1β); Increases IL-10 and adiponectin.	Protects endothelium; Increases eNOS; Reduces LOX-1 expression; Reduces adhesion molecules (ICAM-1, VCAM-1).	Reduces LDL-C, TC, TG; Increases HDL-C; Promotes the leptin-to-adiponectin ratio.
**Fisetin**	Weakly directly activates SIRT1 and Indirectly activates SIRT1 via AMPK activation and increasing SIRT1 expression.	Reduces ROS; Enhances antioxidant enzymes (SOD, GPx, CAT, HO-1); Inhibits oxidative enzymes (NOX); Modulates PKC-δ, p38, and Nrf2-ARE signaling pathways; Inhibits LDL oxidation.	Inhibits Akt, NF-κB and Erk½; Inhibits pro-inflammatory cytokines (TNF-α, IL-6, IL-1β); Inhibits pro-inflammatory genes (MCP-1, iNOS, uPA); Inhibits COX2 and MMP9 expression.	Inhibits platelets aggregation; Inhibits PKC activity; Reduces adhesion molecules (ICAM-1).	Reduces TC, LDL-C, VLDL-C.
**Piceatannol**	Directly activates SIRT1 and also indirectly through upregulating SIRT1 expression.	Reduces ROS; Enhances antioxidant enzymes (HO-1); Promotes Nrf2 activation.	Inhibits NF-κB; Inhibits pro-inflammatory cytokines (TNF-α, IL-6, lkBα); Inhibits COX2 and MMP9 expression.	Protects endothelium; Blocks Erk½, JNK, PI3K/Akt pathways; Inhibits VSMC migration.	Inhibits lipogenesis; Reduces TG; Blocks SREBP-1, ACC, PPARγ; Lowers circulating free fatty acids: downregulates CD36 and induces degradation of ATGL, CGI-58 and PLIN1.
**Honokiol**	Directly activates SIRT1 and indirectly by enhancing SIRT1 expression.	Reduced ROS; Inhibits Fe(III) ADP/NADH; Enhances antioxidant enzymes (SOD, CAT); Enhances Keap1/Nrf2/ARE and GSH; Inhibits LDL oxidation.	Inhibits NF-κB and MAPK; Inhibits pro-inflammatory cytokines (TNF-α, IL-6, IL-1β); Inhibits pro-inflammatory enzymes (COX2, NOS, iNOS);	Protects endothelium; Inhibits TXA_2_ formation; Promotes NO releasing; Reduces adhesion molecules (ICAM-1, VCAM-1).	Reduces LDL-C, TC, TG; Increases HDL-C; Promotes lipophagy through SIRT3/AMPK pathway.
**Epigallocatechin-3-gallate**	Weakly directly activates SIRT1 and Indirectly activates SIRT1 via AMPK activation and enhancing SIRT1 expression.	Reduced ROS; Inhibits oxidative enzymes (NOX); Enhances antioxidant enzymes (SOD, HO-1); Promotes Nrf2 activation.	Inhibits NF-κB and MAPK; Inhibits pro-inflammatory cytokines (TNF-α, CRP); Inhibits TLR4 expression; Binds notch receptors, blocking notch inflammatory cascade.	Protects endothelium; Reduces adhesion molecules (ICAM-1, VCAM-1).	Reduces TG, TC and LDL-C; Increases HDL-C; Downregulates PPARγ and FAS expression; Blocks SREBP-1; Activates the X receptor-mediated cholesterol efflux genes (ABCA1, ABCG5/8 and LXRα/β).
**Hydroxytyrosol**	Indirectly activates SIRT1 by promoting SIRT1 expression.	Reduced ROS; Reduces malondialdehyde (MDA); Activates *AKT*1; Enhances antioxidant enzymes (SOD), HO-1); Promotes Nrf2 activation; Enhances GSH.	Inhibits pro-inflammatory cytokines (TNF-α, IL-6, IL-1β); Inhibits COX_2_ expression.	Improves endothelial function; Increases eNOS; Reduces adhesion molecules (ICAM-1, VCAM-1); decreases E-selectin, P-selectin; Inhibits TX and prostacyclin synthesis.	Regulates reverse cholesterol efflux by increasing ABCA1 expression and activating AMPK pathway and phosphorylation of p38.

**Table 2:** Summary of the human data available for each compound, involving the study design, the number of the subjects recruited, the dosage, the duration of the study, and the primary outcomes.

Compound	Sources	Design	Type/Dose	Population	Primary Outcomes	Limitations
**Resveratrol**	Reviews/meta-analysis and ongoing pilot RCTs (2023–2025).	Multiple RCTs and pilot trials.	Oral resveratrol supplements; High-dose resveratrol (ranging from ~0.5–1.5 g/day).	Elderly or high-risk cardiovascular subjects, metabolic disease cohorts.	Mixed and inconsistent effects on endothelial/vascular biomarkers; some trials show anti-inflammatory or modest metabolic effect [162].	Contradictory results between studies; need larger standardized trials.
**Quercetin**	Trials of quercetin alone and dasatinib + quercetin pilot human trials; recent 2024–2025 trials target senescence and vascular endpoints.	Small RCTs/pilot studies.	Quercetin dosing varies (intermittent high dose in senolytic protocols; or daily supplement doses).	Older adults, patients with CVD.	Early evidence that quercetin reduces senescent cell markers and may improve physical/cognitive function or vascular biomarkers in pilot studies [163,164].	Small pilot trials; combined drug effects dasatinib + quercetin make complicate to attribute the effects; long term outcomes and safety need larger trials.
**Naringenin**	Systematic reviews and recent pilot clinical studies through 2024–2025; preclinical to pilot human data on blood pressure and metabolic effects.	Mostly pilot human studies and many preclinical studies; a few small RCTs/pilot interventions reported.	Oral supplementation; doses variable depending on extract form short durations in pilot.	Hypertensive models, metabolic syndrome models, human pilot cohorts.	Preclinical and pilot data show antihypertensive and cardioprotective signals; some human pilot studies report improved vascular biomarkers [165].	Human data still limited vs. strong preclinical literature; small sample sizes and short follow-up; dosing/formulation heterogeneity.
**Curcumin**	Multiple 2023–2025 meta-analyses and RCTs.	RCTs and pooled analyses.	Different formulations (standard and enhanced bioavailability).	Obese patients with type 2 diabetes; other at-risk groups.	Improvement in atherogenic risk markers, lipids and inflammatory biomarkers [166,167].	High heterogeneity of formulations/doses; many trials are short; variable quality across trials.
**Berberine**	Multiple meta-analyses and pilot trial (2023–2025).	Randomized, double-blind trials and many small RCTs.	Oral formulations, generally 0.5–1.5 g/day.	Adults with type 2 diabetes/metabolic disorders.	Improvements in cardiometabolic parameters; improvement in lipid profile [168,169].	Trial often targets metabolic endpoints rather than direct atherosclerosis hallmarks; trial formulations vary limiting comparability.
**Fisetin**	Pilot/translational clinical trials (2023–2025); intermittent fisetin vascular trials.	Small pilot RCTs/early human trials.	Intermittent high-dose regimens used in pilot senolytic protocols (days of dosing repeated monthly).	Older adults/healthy volunteers/vascular aging cohorts.	Signals of reduced markers of cellular senescence, improved vascular function or surrogate measures in early trials [107,170,171].	Mostly pilots are in early phase; small number of participants; surrogate endpoints rather than hard CV outcomes.
**Piceatannol**	Limited cardiovascular RCTs: randomized, placebo-controlled study.	39 subjects, 19 (BMI ≥ 25), 20 (BMI < 25).	Early PK studies.	20 mg/day or placebo capsules for 8 weeks.	CV endpoints not relevant [172].	Small sample size; effect only in overweight men; short duration; surrogate markers.
**Honokiol**	Very limited controlled human data.	Small and heterogenous cohorts.	Magnolia extracts (variable honokiol content).	Short pilot exposures/traditional use.	Human CV efficacy data lacking; human safety data limited [173].	Limited data on safety/tolerability in humans.
**EGCG**	Randomized, double-blind, placebo-controlled trials; meta-analyses (2023–2025).	Small to moderate sample sizes (60–200 per trial).	Dose ranging widely (up to 800 mg/day).	Obese subjects, metabolic disease cohorts.	Modest reduction in ratio LDL/TC, reduction in TG; modest improvements in blood pressure and metabolic markers [174,175].	Heterogeneity of doses; some trials are small and short; variable formulations.
**Hydroxytyrosol**	Pilot RCTs and randomized nutritional intervention studies (2023–2025).	Pilot randomized trials (parallel or crossover).	EVOO enriched with hydroxytyrosol or purified hydroxytyrosol.	Healthy older adults, overweight/prediabetes; post myocardial infarction in elderly.	Improvements in antioxidant markers and HDL functions, improved endothelial markers in some studies. Safe at typical intake levels [176,177].	Often small, short trials’ dietary background (other olive oil components); surrogate endpoints.

## Data Availability

The review is based exclusively on previously published studies. No new raw data were generated. Literature was identified through systematic searches of PubMed and Scopus databases, using combinations of the terms “*SIRT1*”, “nutraceuticals”, “polyphenols”, “flavonoids”, “atherosclerosis”, and “vascular protection”. The search covered publications from January 2000 to June 2025, restricted to English-language. Both in vitro, in vivo, and human clinical studies were considered. Screening criteria included the following: (I) relevance to *SIRT1* activity or modulation, (II) evidence of cardiovascular or atherosclerosis-related outcomes, and (III) studies involving the selected nutraceuticals (resveratrol, quercetin, naringenin, curcumin, berberine, fisetin, piceatannol, honokiol, EGCG, and hydroxytyrosol). Exclusion criteria included being unrelated to cardiovascular or *SIRT1* outcomes. The study selection process is summarized in the PRISMA flow diagram (Figure 1). All data supporting the findings of this review are available in the cited publications.

## Abbreviations

The following abbreviations are used in this manuscript:

ABCA1	ATP-binding cassette transporter A1
*ACAT*	Hepatic cholesterol acyltransferase
*ACC*	Acetyl-CoA carboxylase
*Akt*	Protein kinase B
*AMPK*	Adenosine monophosphate-activated kinase
ApoB	ApolipoproteinB
ARE	Antioxidant response element
*ATGL*	Adipose triglyceride lipase
BBR	Berberine
BKCa	Calcium-activated potassium channel
cAMP	Cyclic adenosine monophosphate
*CAT*	Catalase
CCL2	Chemokine (C-C motif) ligand 2
CGI-58	Comparative gene identification-58
*COX-2*	Cyclooxygenase-2
CRC	Curcumin
CRP	C-reactive protein
CVD	Cardiovascular disease
CXCL1	Chemokine (C-X-C motif) ligand 1
EGCG	Epigallocatechin-3-gallate
*eNOS*	Endothelial nitric oxide synthase
*EP300*	E1A binding protein p300 acetyltransferase
*Erk*	Extracellular signal-regulated kinases
*FAS*	Fatty acid synthase
FIS	Fisetin
FOXO	Forkhead box-O
Gal-3-NLRP3	Galectin-3 NLR family pyrin domain containing 3
*GPx*	Glutathione peroxidase
GSH	Glutathione
HCAEC	Human coronary artery endothelial cell
HDL	High-density lipoprotein
HDL-C	High-density lipoprotein cholesterol
*HIF-1α*	Hypoxia-inducible factor 1-alpha
HMGB1	High Mobility Group Box 1
HNK	Honokiol
*HO-1*	Heme oxygenase-1
HOCl	Hypochlorous acid
HT	Hydroxytyrosol
ICAM-1	Intercellular adhesion molecule-1
Il-1β	Interleukin-1β
Il-6	Interleukin-6
Il-8	Interleukin-8
*iNOS*	Inducible nitric oxide synthase
*IκBα*	Nuclear factor of kappa light polypeptide gene enhancer in B-cells inhibitor alpha
*JNK*	c-Jun N-terminal kinases
KL	Klotho
LDL	Low-density lipoprotein
LDL-C	Low-density lipoprotein cholesterol
LDLR	Low-density lipoprotein receptor
*LKB1*	Liver kinase B1
*LOX*	Lipoxygenases
LXRα	Calmodulin-liver X receptor α
MAP1LC3	Microtubule-associated protein 1 light chain 3
*MAPK*	Mitogen activated protein kinase
MCP-1	Monocyte chemo attractant protein-1
MDA	Malondialdehyde
*MMP-9*	Metalloproteinase-9
*Mn-SOD*	Manganese superoxide dismutase
*MPO*	Myeloperoxidase
mTOR	Mammalian target of rapamycin
mTORC1	Mammalian target of rapamycin complex 1
NAD	Nicotinamide adenine dinucleotide
NADPH	Nicotinamide adenine dinucleotide phosphate
NAR	Naringenin
*NF-κB*	Nuclear factor kappa B
NLRP3	Nucleotide-binding oligomerization domain-like receptor protein 3
NO	Nitric oxide
*NOX*	Nicotinamide adenine dinucleotide phosphate oxidase
*Nrf2*	Nuclear factor erythroid 2-related factor 2
oxLDL	Oxidized low-density lipoprotein
PCT	Piceatannol
*PGC-1α*	Peroxisome proliferator-activated receptor gamma coactivator 1-alpha
PGE_2_	Prostaglandin E_2_
*PI3K*	Phosphoinositide 3-kinase
*PKA*	Protein kinase A
*PKC-δ*	Protein kinase C-delta
PLIN1	Perilipin 1
*PPARγ*	Peroxisome proliferator-activated receptor gamma
Prx	Peroxiredoxin
QRC	Quercetin
RASMC	Rat aortic smooth muscle cells
RCT	Reverse cholesterol transport
RNS	Reactive nitrogen species
ROS	Reactive oxygen species
RSV	Resveratrol
*SIRT1*	Sirtuin 1
*SOD*	Superoxide dismutase
SR-A1	Scavenger receptor class A
*SREBP-1*	Sterol regulatory element-binding transcription factor 1
TC	Total cholesterol
*TF*	Tissue factor
*TFEB*	Transcription factor EB
TG	Triglycerides
TLR4	Toll-like receptor 4
TMA	Trimethylamine
TMAO	Trimethylamine N-oxide
*TNFα*	Tumor necrosis factor α
*TR2*	Thioredoxin reductase 2
Trx2	Thioredoxin 2
TSC2	Tuberous Sclerosis Complex 2
TXA_2_	Thromboxane A_2_
uPa	Urokinase plasminogen activator
UVEC	Umbilical vein endothelial cells
VCAM-1	Vascular adhesion molecule-1
VSMC	Vascular smooth muscle cells
*XO*	Xanthine oxidases
